# Whole abdomen radiation therapy in ovarian cancers: a comparison between fixed beam and volumetric arc based intensity modulation

**DOI:** 10.1186/1748-717X-5-106

**Published:** 2010-11-15

**Authors:** Umesh Mahantshetty, Swamidas Jamema, Reena Engineer, Deepak Deshpande, Rajiv Sarin, Antonella Fogliata, Giorgia Nicolini, Alessandro Clivio, Eugenio Vanetti, Shyamkishore Shrivastava, Luca Cozzi

**Affiliations:** 1 Radiation Oncology Department, Tata Memorial Centre (TMC), Mumbai, India; 2 Radiation Oncology Department, Oncology Institute of Southern Switzerland, Bellinzona, Switzerland

## Abstract

**Purpose:**

A study was performed to assess dosimetric characteristics of volumetric modulated arcs (RapidArc, RA) and fixed field intensity modulated therapy (IMRT) for Whole Abdomen Radiotherapy (WAR) after ovarian cancer.

**Methods and Materials:**

Plans for IMRT and RA were optimised for 5 patients prescribing 25 Gy to the whole abdomen (PTV_WAR) and 45 Gy to the pelvis and pelvic nodes (PTV_Pelvis) with Simultaneous Integrated Boost (SIB) technique. Plans were investigated for 6 MV (RA6, IMRT6) and 15 MV (RA15, IMRT15) photons. Objectives were: for both PTVs V_90% _> 95%, for PTV_Pelvis: D_max _< 105%; for organs at risk, maximal sparing was required. The MU and delivery time measured treatment efficiency. Pre-treatment Quality assurance was scored with Gamma Agreement Index (GAI) with 3% and 3 mm thresholds.

**Results:**

IMRT and RapidArc resulted comparable for target coverage. For PTV_WAR, V_90% _was 99.8 ± 0.2% and 93.4 ± 7.3% for IMRT6 and IMRT15, and 98.4 ± 1.7 and 98.6 ± 0.9% for RA6 and RA15. Target coverage resulted improved for PTV_Pelvis. Dose homogeneity resulted slightly improved by RA (Uniformity was defined as U_5-95% _= D_5%_-D_95%_/D_mean_). U_5_-_95% _for PTV_WAR was 0.34 ± 0.05 and 0.32 ± 0.06 (IMRT6 and IMRT15), 0.30 ± 0.03 and 0.26 ± 0.04 (RA6 and RA15); for PTV_Pelvis, it resulted equal to 0.1 for all techniques. For organs at risk, small differences were observed between the techniques. MU resulted 3130 ± 221 (IMRT6), 2841 ± 318 (IMRT15), 538 ± 29 (RA6), 635 ± 139 (RA15); the average measured treatment time was 18.0 ± 0.8 and 17.4 ± 2.2 minutes (IMRT6 and IMRT15) and 4.8 ± 0.2 (RA6 and RA15). GAI_IMRT6 _= 97.3 ± 2.6%, GAI_IMRT15 _= 94.4 ± 2.1%, GAI_RA6 _= 98.7 ± 1.0% and GAI_RA15 _= 95.7 ± 3.7%.

**Conclusion:**

RapidArc showed to be a solution to WAR treatments offering good dosimetric features with significant logistic improvements compared to IMRT.

## Introduction

Epithelial Ovarian Cancer (EOC) is a malignancy with significant probability of trans-peritoneal diffusion for which, irrespective of multiple surgeries and chemotherapy applications, a recurrence rate of 60-70% has been reported [1]. Though not a standard treatment, Whole Abdomen Radiotherapy (WAR), as adjuvant or as salvage approach has been attempted with limited success [2,3]. The target volume for WAR includes the whole of abdominal-pelvic cavity with all its contents. Though effective in principle, WAR is technically challenging because of inadequate coverage of large target volume and poor sparing of organs at risk (OAR) with risk of severe toxicity [4,5].

The easiest approach to irradiate such a target is to use simple anterior/posterior beam arrangement with partial kidney and liver protection [6,7].

The application of advanced techniques like Intensity Modulated Radiotherapy (IMRT) or Intensity Modulated Arc Therapy (IMAT) has shown potential to achieve sufficient uniformity to the target with improved sparing of OARs [8-10]. WAR with Helical Tomotherapy (HT) was investigated by Rochet et al [11,12] with a fractionation scheme of 30 Gy in 1.5 Gy per fraction to the entire peritoneal cavity including pelvis and para-aortal node regions without pelvic boost. Similar approach was followed by Hong et al [8] and by Duthoy et al [9] (in the latter case with a prescription of 33 Gy). Garsa et al [10] applied also a boost up to 44.4 Gy to the pelvis. Other prescription of radiation doses between 20 and 30 Gy over 20 -25 fractions for WAR and a boost between 40.4 and 51 Gy has been reported [13-15]. This wide dose range is mostly due to the absence of a well defined dose response relationship and of an established consensus till date. However, doses higher than 30 Gy to whole abdomen and pelvic doses >50 Gy are associated with higher incidence of small bowel toxicities [15] requiring surgery. Moreover, studies which have shown a substantial benefit in progression free and overall survivals have had a component of pelvic boost in abdominal-pelvic radiation protocols [13,14]. In addition, although the concept of pelvic boost might be considered as controversial and its routine usage in consolidation WAR might be questioned [16], the pelvis is the major site of relapse and higher doses are tolerated by the pelvis [11,17]. It is therefore interesting to investigate how it can be managed with a Simultaneous Integrated Boost (SIB) fractionation scheme and intensity modulation. As in Jamema et al [18], in this study a SIB approach will be investigated for feasibility aiming to be proposed to patients with complete response after surgery and chemotherapy or with minimal residual disease (< 1 cm) as a consolidation therapy.

From the technology point of view, IMAT recently evolved to the concept of volumetric delivery. RapidArc (RA), is a method to deliver volumetric intensity-modulated arc therapy based on the original investigations of Otto [19] and was adopted for this investigation. A detailed description of the principles of RapidArc can be found in [20]. RapidArc has been investigated, compared to IMRT or other approaches, in a series of studies including brain tumors, prostate, head and neck, anal canal, cervix uteri and other indications [21-28] showing some general features summarized in: i) tendency to improve sparing of organs at risk with respect to conventional, IMRT and sometimes more advanced approaches; ii) tendency to achieve similar or slightly improved target coverage with respect to IMRT; iii) reduced delivery times and lower number of monitor units per Gy (compared to IMRT).

Purpose of the present work was to further investigate RapidArc spectrum of possible clinical applications with a feasibility study. Benchmark for reference was chosen to be fixed gantry intensity modulation technique, widely available in most of the clinics. This purpose was assessed by measuring i) the capability of RapidArc to generate high quality dose distributions with homogeneous doses to WAR target and high sparing of kidneys, bone marrow and liver; ii) the logistic aspects of treatment efficiency; iii) the degree of agreement of delivered vs. computed dose distributions. Benchmark used for this analysis is ''conventional'' fluence-based fixed gantry IMRT.

## Methods and Materials

**Figure 1:**
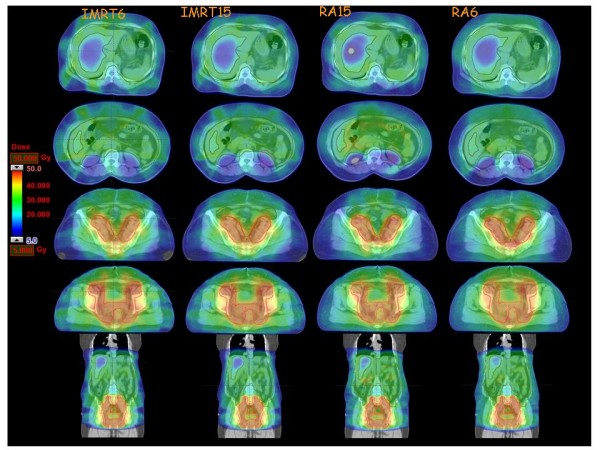
**Isodose distributions in four axial and a coronal views for one example case**. Color wash is cut between 5 Gy and 50 Gy. Also shown the overlay of PTV and main organs at risk.

CT datasets of five patients were used for this study. Being a planning study aiming to a feasibility demonstration, data were collected from patients with recurrent epithelial ovarian cancers of post operative gynecological cancer, for this reason no clinical details of the patients are provided. CT Scans were performed with patients in supine position, arms above head. All patients were asked to drink 500-1000 ml of water, after having emptied bladder, 45 minutes prior to CT scan for constant moderate bladder filling. CT scans extended from the mid thorax to mid thighs with 5 mm contiguous slice thickness. Abdominal Clinical Target Volume (CTV_WAR) included entire peritoneal cavity with bowel and mesentery, liver capsule with surface of liver parenchyma, under surface of liver, abdominal surface of diaphragm and anterior-lateral surfaces of both the kidneys. Pelvic CTV (CTV_Pelvis) was contoured from L5-S1 vertebral junction to include pelvic lymph nodal regions, pouch of Douglas and vaginal vault. Abdominal Planning Target Volume (PTV_WAR) was drawn with differential margins to CTV, 1.5 cm cranially (for diaphragm movements) and 0.5 cm in all other directions, similarly pelvic PTV (PTV_Pelvis) was drawn with 1 cm margin in caudal direction and 0.5 cm margin in all other directions. Organs at risk (OAR) included: kidneys, liver, bone marrow (ribs, vertebrae, pelvic bones and upper end femora), bladder, rectum and heart. A structure named "normal liver" was created just inside liver to control doses within liver parenchyma not included in PTV_WAR (the width of the outer liver border included in the PTV_WAR ranged from 1 to 1.5 cm). Also for kidneys the same 1-1.5 cm of rim was included in the PTV_WAR, and results will be reported for the entire kidneys or for the fraction of kidney outside PTV. This margin was defined to allow adequate treatment of capsule and rim of OARs parenchyma with margins for movements. PTV never reached the surface of the body patients (as can be seen as an example in figure 1).

### Treatment Planning

A total dose of 25 Gy in 25 fractions with 1 Gy per fraction was prescribed to PTV_WAR with simultaneous boost of 45 Gy in 25 fractions at 1.8 Gy per fraction to PTV_Pelvis. Plan normalization was set to mean dose to PTV_Pelvis. Assuming an α/β ratio of 10, the dose prescription to PTV_WAR corresponds to a Biological Equivalent Dose BED of 27.5 Gy (in 25 fractions). This prescription, lower than what reported in other studies (e.g. [11,12]) is based on: i) institutional clinical practice, ii) trade-off against toxicity on major organs at risk, iii) presence of a simultaneous boost on the pelvis, major site of recurrences.

Two sets of plans were compared in this study, all designed by the same planner on the Varian Eclipse treatment planning system (TPS) (version 8.6) with photon beams from a Varian Clinac equipped with a Millennium Multileaf Collimator (MLC) with 120 leaves (spatial resolution of 5 mm at isocentre for the central 20 cm and of 10 mm in the outer 2 × 10 cm, leaf transmission of 1.8%). Plans for RapidArc were optimised selecting a maximum DR of 600 MU/min and a fixed DR of 600 MU/min was selected for IMRT.

The Anisotropic Analytical Algorithm (AAA) photon dose calculation algorithm was used for all cases [29]. The dose calculation grid was set to 2.5 mm.

The general strategy adopted for both IMRT and RapidArc was to use two isocentres, aligned in x and y and separated only in z (cranial-caudal direction of the patient) of about 15 cm. Rationale for this choice was the length of the total PTV which ranged from 45 to 51 cm. The upper and lower fields or arc overlap by about 5 cm. The same isocentre settings were used for IMRT and RapidArc plans. In both cases, all fields or arcs were simultaneously optimised to generate the desired dose distributions on all targets.

#### IMRT

The dynamic sliding window fluence-based method with fixed gantry beams was used [30,31] as reference benchmark. A total of fourteen beams with fixed jaws settings were applied grouped in two sets of 7 beams per each isocentre at 0°, 51°, 102°, 153°, 207°, 258°, 309°. Beam angles were selected in order to avoid opposite entrance. All beams were coplanar with collimator angle set to 0°. The first group of beams covered primarily PTV_WAR and the second group PTV_Pelvis. No bolus was applied. A high smoothing factor was applied during optimisation (with the same priority of the highest priority used for dose volume objectives) to minimise the MU/Gy from IMRT.

#### RapidArc (RA)

RapidArc uses continuous variation of the instantaneous dose rate (DR), MLC leaf positions and gantry rotational speed to optimise the dose distribution [21,22]. A collimator angle different from 0° is used to smear residual tongue-and-groove and interleaf leakage effects in non planar trajectories and, more important, to allow transverse spatial modulation [20] per each degree and per each axial section of the patient.

Plans were optimised with two arcs of 360° each and a third arc of 280° excluding the posterior sector (being RapidArc optimiser in Eclipse 8.6 limited to a total gantry rotation of 1000°). The first two arcs, rotating clock- and counter-clock-wise to minimise dead time between the two when delivered, were incident primarily on PTV_WAR and on the upper part of PTV_Pelvis. The third arc rotating, was incident primarily on PTV_Pelvis and on the caudal part of PTV_WAR. The overlap between the two group of arcs was set to ~5 cm. In the present study the collimator was rotated to ± 30° for the two arcs irradiating PTV_WAR and to 45° for the arc covering PTV_Pelvis. The rationale to use two arcs for PTV_WAR was the huge volume of the target volume and the need of high sparing of OARs almost embedded inside PTVs (liver and kidneys). A multiple arc in this case is expected to enhance the homogeneity of the dose and to increase the sparing potential of OARs.

For both techniques, RapidArc and IMRT, two sets of plans were optimised for each patient using beams of nominal energy of either 6 MV or 15 MV. At higher energies it is expected to achieve the better homogeneity with targets of the sizes involved in this study.

Planning objectives for both PTVs aimed to maximise coverage with V_90% _> 95%, for pelvic PTV maximum dose was constrained to D_2% _< 47.3 Gy (105% of prescription dose) and V_107% _< 1% (where V_x% _are target volumes receiving at least x% of the prescribed dose as D_x% _are dose levels delivered at least to x% of volumes). The constraints to D_2% _and V_107% _were not applied to PTV_WAR since it directly abutted with PTV_Pelvis; in this case D_2% _and V_107% _were to be minimised. In addition, on targets, dose homogeneity was aimed to be enhanced as much as possible. For organs at risk, plans were optimised to obtain the maximum sparing achievable without severe violations of PTV coverage. The MU and delivery time measured treatment efficiency.

### Evaluation Parameters

Evaluation of plans was based on Dose-Volume Histogram (DVH) analysis. For PTV, the values of D_98% _and D_2% _(dose received by the 98, and 2% of the volume) were defined as metrics for minimum and maximum doses. Also V_90% _V_95% _V_107% _(the volumes receiving at least 90%, 95%, 107% of the prescribed dose) were reported. The homogeneity of the dose distribution, was measured by D_5%_-D_95% _and expressed as U_5-95% _= D_5%_-D_95%_/D_mean_. The lower this value, the better is the dose homogeneity. Equivalent Uniform Dose (EUD) was computed as well. Conformity Index, CI_95%_, was defined as the ratio between the patient volume receiving at least 95% of the prescribed dose and the volume of the total PTV_Pelvis, measured the conformity of the high dose levels. For OARs, the analysis included the mean dose, the maximum dose expressed as D_2% _and a set of V_XGy _(OAR volume receiving at least x Gy) depending upon the organ. For Healthy Tissue (defined as the entire body volume included in the CT scan minus the PTVs), the integral dose, "DoseInt" was defined as the integral of the absorbed dose extended to over all voxels excluding those within the target volume (DoseInt dimensions are Gy*cm^3^). This was reported together with the observed mean dose and V_10 Gy_.

Average cumulative DVH for PTV, OARs and healthy tissue, were built from the individual DVHs for qualitative visualisation of results. These histograms were obtained by averaging the corresponding volumes over the whole patient's cohort for each dose bin of 0.05 Gy.

Delivery parameters were recorded in terms of MU per fraction, beam on time and treatment time (defined as beam-on plus machine programming and setting time and excluding patient positioning and imaging procedures).

Pre-treatment quality assurance results were summarised in terms of the Gamma Agreement Index, GAI, scoring the percentage of modulated area fulfilling the γ index criteria [32] (computed with 2 and 3% and 2 and 3 mm thresholds). The software utilised to analyse dosimetric data was Epiqa (Epidos sro, Slovakia) based on the GLAaS algorithm developed by authors [33,34]. Pre-treatment dosimetry was considered satisfactory if GAI exceeded 95%.

The Wilcoxon matched-paired signed-rank test was used to compare the results. The threshold for statistical significance was *p *≤ 0.05. All statistical tests were two-sided.

## Results

**Figure 2:**
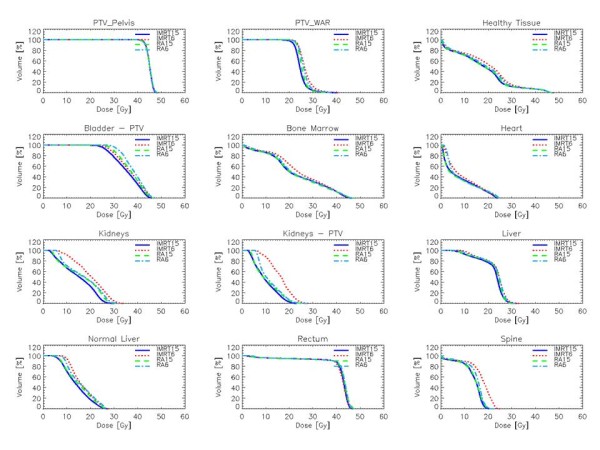
**Mean dose volume histograms for PTVs and main organs at risk**.

Dose distributions are shown for one patient in Figure 1 for four different axial planes and a coronal view. Colour wash banding is restricted to 5-50 Gy. Figure 2 shows the average DVH for the targets and the organs at risk. Table 1 reports numerical findings from DVH analysis on PTV_WAR, PTV_Pelvis and Healthy Tissue, Table 2 on various OARs. Data are presented as averages over the five investigated patients and errors indicated inter-patient variability at 1 standard deviation level. Statistical significance is reported when p < 0.05 according to description in the foot notes of the tables.

**Table 1:** Summary of DVH analysis for PTV_A, PTV_Pelvis and healhty tissue.

	Objective	IMRT6	IMRT15	RA15	RA6	p
				**PTV_WAR **(6524 ± 861 cm^3^)		
**Mean [Gy]**	25.0 Gy	26.8 ± 0.5	24.8 ± 0.9	25.5 ± 0.5	26.2 ± 0.7	a,c,d
**EUD [Gy]**	-	25.9 ± 0.4	24.0 ± 1.0	25.0 ± 0.3	25.6 ± 0.7	a,c,d
**D_2% _[Gy]**	Minimise	35.1 ± 1.4	32.9 ± 2.7	31.5 ± 2.2	33.2 ± 2.6	a,b,c,d
**U_5-95%_**	Minimise	0.34 ± 0.05	0.32 ± 0.06	0.26 ± 0.04	0.30 ± 0.03	a,b,d
**D_98% _[Gy]**	> 22.5 Gy	22.3 ± 0.5	20.7 ± 1.4	21.9 ± 0.4	22.1 ± 0.9	a,c
**V_90% _[%]**	> 95%	99.0 ± 0.6	93.4 ± 7.3	98.6 ± 0.9	98.4 ± 1.7	a,b,c
**V_95% _[%]**	-	95.6 ± 1.6	89.5 ± 12.0	92.7 ± 2.4	94.1 ± 5.0	a,b,c,d
**V_107% _[%]**	Minimize	36.3 ± 7.7	13.7 ± 7.7	17.6 ± 8.2	20.1 ± 11.6	a,b,c,d

				**PTV_Pelvis **(1211 ± 158 cm^3^)		
**Mean [Gy]**	45.0 Gy	45.0 ± 0.0	45.0 ± 0.0	45.0 ± 0.0	45.0 ± 0.0	-
**EUD [Gy]**	-	44.0 ± 1.6	44.0 ± 1.5	43.9 ± 1.6	44.0V1.9	NS
**D_2% _[Gy]**	< 47.3 Gy	47.6 ± 0.5	47.3 ± 0.4	47.5 ± 0.3	47.5 ± 0.2	NS
**U_5-95%_**	Minimise	0.1 ± 0.02	0.10 ± 0.01	0.10 ± 0.02	0.10 ± 0.02	NS
**D_98% _[Gy]**	> 40.5 Gy	42.2 ± 0.4	42.1 ± 0.5	41.7 ± 0.5	41.7 ± 0.2	NS
**V_90% _[%]**	> 95%	99.8 ± 0.2	99.7 ± 0.4	99.5 ± 0.3	99.5 ± 0.2	NS
**V_95% _[%]**	-	95.4 ± 1.9	95.2 ± 2.3	94.0 ± 2.1	94.2 ± 1.1	NS
**V_107% _[%]**	< 2%	0.7 ± 0.9	0.3 ± 0.6	0.5 ± 0.6	0.5 ± 0.6	NS
**CI_95%_**	minimise	1.60 ± 0.1	1.51 ± 0.1	1.50 ± 0.1	1.50 ± 0.1	NS

				**Healthy tissue **(25772 ± 7089 cm^3^)		
**Mean [Gy]**	-	19.6 ± 2.1	17.6 ± 1.3	17.6 ± 1.5	18.6 ± 1.6	b,c
**V_10 Gy _[%]**	-	75.0 ± 6.7	71.1 ± 5.4	70.4 ± 5.1	72.8 ± 5.5	b,c
**DoseInt [Gy cm^3 ^10^6^]**	-	4.94 ± 0.90	4.48 ± 1.00	4.48 ± 0.92	4.74 ± 1.02	b.c

D_x% _= dose received by the x% of the volume; V_x% _= volume receiving at least x% of the prescribed dose; CI = ratio between the patient volume receiving at least 95% of the prescribed dose and the volume of the total PTV. U_5-95% _= D_5_-D_95_/D_mean _EUD = Equivalent Uniform Dose. DoseInt = Integral dose, [Gy cm^3 ^10^6^]. Statistical significance (p): a = IMRT15 vs RA15, b = IM6 vs RA6, c = IMRT15 vs IMRT6, d = RA15 vs RA6, NS = Not significative

**Table 2:** Summary of DVH analysis for organs at risk

	IMRT6	IMRT15	RA15	RA6	P
			**Bone Marrow **(1095 ± 235 cm^3^)		
**Mean [Gy]**	24.7 ± 1.4	22.8 ± 1.5	22.6 ± 1.5	23.4 ± 1.3	b,c
**D_1% _[Gy]**	44.9 ± 0.9	44.8 ± 0.4	45.5 ± 0.7	45.6 ± 0.9	a,b
**V_30 Gy _[%]**	35.0 ± 4.0	31.8 ± 4.1	29.6 ± 5.1	31.5 ± 5.1	b,c
**V_45 Gy _[%]**	1.3 ± 1.2	0.9 ± 0.7	2.2 ± 1.3	2.2 ± 1.4	a,b

			**Bladder **(208 ± 219 cm^3^)		
**Mean [Gy]**	39.6 ± 1.9	38.7 ± 2.6	39.5 ± 1.5	40.4 ± 1.2	NS
**D_1% _[Gy]**	46.9 ± 0.8	48.5 ± 15.2	46.5 ± 0.5	46.9 ± 0.7	NS
**V_40 Gy _[%]**	57.8 ± 11.3	58.4 ± 25.8	56.7 ± 9.6	61.4 ± 9.1	NS

			**Bladder - PTV **(131 ± 140 cm^3^)		
**Mean [Gy]**	36.5 ± 1.8	35.2 ± 0.8	36.9 ± 0.9	38.3 ± 0.8	a,b,d
**D_1% _[Gy]**	45.1 ± 1.3	44.7 ± 1.0	45.3 ± 1.0	46.0 ± 1.6	b
**V_40 Gy _[%]**	32.7 ± 12.5	26.1 ± 7.1	31.9 ± 8.6	39.6 ± 8.3	NS

			**Heart **(438 ± 166 cm^3^)		
**Mean [Gy]**	9.1 ± 1.6	7.8 ± 1.0	7.7 ± 1.0	8.6 ± 1.1	c,d
**D_1% _[Gy]**	23.8 ± 0.5	23.4 ± 0.2	24.2 ± 0.4	24.5 ± 0.4	a,b

			**Kidneys **(242 ± 21 cm^3^)		
**Mean [Gy]**	20.0 ± 3.3	14.7 ± 1.8	16.0 ± 1.8	17.0 ± 2.0	a,b,c
**D_1% _[Gy]**	31.5 ± 1.5	27.3 ± 1.5	29.7 ± 1.3	29.6 ± 1.5	a,b,c
**V_20 Gy _[%]**	51.7 ± 14.7	32.1 ± 8.0	41.1 ± 9.5	42.0 ± 11.0	c

			**Kidneys - PTV **(141 ± 18 cm^3^)		
**Mean [Gy]**	16.1 ± 3.0	9.8 ± 1.9	10.3 ± 0.9	11.7 ± 0.7	b,c
**D_1% _[Gy]**	25.7 ± 1.9	20.9 ± 1.2	23.2 ± 1.0	23.9 ± 1.5	a,b,c
**V_20 Gy _[%]**	22.9 ± 11.9	2.7 ± 28	5.7 ± 2.2	6.9 ± 2.5	a,b,c,d

			**Liver **(1144 ± 231 cm^3^)		
**Mean [Gy]**	23.2 ± 0.5	21.8 ± 0.9	22.4 ± 0.7	22.9 ± 0.3	NS
**D_1% _[Gy]**	30.9 ± 1.0	29.4 ± 1.4	28.7 ± 0.9	29.6 ± 0.9	NS
**V_20 Gy _[%]**	80.7 ± 2.6	77.1 ± 3.5	79.5 ± 3.2	80.5 ± 2.3	c

			**Normal Liver **(273 ± 84 cm^3^)		
**Mean [Gy]**	16.4 ± 0.4	14.0 ± 0.9	14.8 ± 0.5	15.9 ± 0.4	b,c
**D_1% _[Gy]**	26.5 ± 0.7	25.2 ± 1.3	25.7 ± 0.8	26.6 ± 0.4	c,d
**V_20 Gy _[%]**	26.1 ± 3.9	17.2 ± 3.8	23.2 ± 3.1	27.9 ± 2.7	a,c,d

			**Rectum **(129 ± 84 cm^3^)		
**Mean [Gy]**	40.7 ± 2.6	40.8 ± 2.4	41.1 ± 2.5	41.3 ± 2.5	NS
**D_1% _[Gy]**	46.1 ± 0.9	45.9 ± 0.7	46.4 ± 0.7	46.6 ± 0.5	NS
**V_45 Gy _[%]**	9.7 ± 10.9	8.2 ± 4.5	14.9 ± 11.7	17.1 ± 6.1	a,b

			**Spine **(23 ± 6 cm^3^)		
**D_1% _[Gy]**	22.8 ± 1.9	19.0 ± 1.8	19.4 ± 1.1	19.6 ± 1.5	b,c

D_x% _= dose received by the x% of the volume; V_x% _= volume receiving at least xGy of the prescribed dose.

Additional file 1: Table S1 presents a synoptic comparison between this and previous investigations on the same subject.

### Target coverage and dose homogeneity

Data summarised in table 1 show that, for PTV_WAR, RA plans are very similar although higher energy (15 MV) could be preferable to lower (6 MV) in terms of lower maximum doses (D_2% _and V_107%_) and better homogeneity (U_5-95%_). RA15 achieved the best uniformity of dose distributions also in terms of EUD while showed the largest deviation with about 1 Gy below (above) prescription for IMRT15 (IMRT6). Concerning PTV_Pelvis, all four groups resulted to be equivalent.

### Organs at risk and healthy tissue

In general, RA15 and IMRT15 resulted in better OARs sparing compared to RA6 and IMRT6, with a tendency to significance of observed differences, suggesting a better role of higher energy in protecting organs at risk. IMRT6 resulted in particular defective in sparing kidneys (and spinal cord) compared to the other techniques. Also for Healthy Tissue, RA6 and IMRT6 showed the worst results compared to both RA15 and IMRT15, suggesting some relevance in using higher energies to better focalise dose and limiting the dose bath.

The comparison between IMRT15 and RA15, suggests a basic equivalence between the two techniques although for bladder-PTV (the portion not included in the PTVs) and Normal Liver, IMRT has a better sparing in the dose levels higher than ~20 Gy.

### Delivery parameters

The number of MU per fraction of 1.8 Gy resulted 3103 ± 221 (IMRT6), 2841 ± 318 (IMRT15), 538 ± 29 (RA6), 635 ± 139 (RA15); this corresponds to the ratios: MU_IMRT15_/MU_RA15 _= 4.6 ± 0.9, MU_IMRT6_/MU_RA6 _= 5.8 ± 0.7.

The total treatment time from load of patient data into treatment console to the end of last delivery, was 4.8 ± 0.2 minutes (220 seconds of beam-on) for RA6 and RA15 compared to 18.0 ± 0.8 for IMRT6 and 17.4 ± 0.8 for IMRT15. This difference, besides MU ratio, is mostly due to the need to re-program the linac between fixed gantry beams, rotate the gantry from one position to the next and to deliver split fields (in average fields were split because of the size of the target). Time to move between isocentres is the same for the two approaches. As mentioned, these values do not include any imaging or patient positioning procedure, common to any technique and not relevant for the comparison.

### Pre-treatment dosimetric measurements

The Gamma Agreement Index, scored with 3 mm and 3% thresholds was: GAI_IMRT6 _= 97.3 ± 2.6%, GAI_IMRT15 _= 94.4 ± 2.1%, GAI_RA6 _= 98.7 ± 1.0%, GAI_RA15 _= 95.7 ± 3.7%. The time (in minutes) needed to perform the pre-treatment QA is: time for data preparation, including field by field calculations: 21.0 ± 1.4 for RA and 14.5 ± 2.1 for IMRT; time for measurements was 3.7 ± 0.3 for RA and 13.4 ± 0.1 for IMRT; time for analysis of data: 1.2 ± 0.1 for RA and 5.1 ± 0.6 for IMRT leading to a total time for QA procedures of: 25.6 ± 1.5 minutes for RA with two arcs and 33.0 ± 1.5 minutes for IMRT with 9 split fields.

## Discussion

WAR in Epithelial Ovarian Cancer, though effective, is used sparingly due to the concerns regarding inadequate coverage of large target volume, and poor sparing of organs at risk leading to significantly higher toxicities [4,5]. Traditionally, conventional radiotherapy techniques using AP/PA fields and 6-15 MV photon beams with partial OAR shielding is applied but this has major dosimetric limitations [6,35]. The advent of newer radiation techniques allowed the possibility to treat large multiple targets with simultaneous integrated boosts with optimal sparing of critical structures.

Three objectives were set for this study. The first was the assessment of the RapidArc capability to generate adequate dose distributions adequate. This result was achieved and RA plans resulted basically equivalent to the benchmark IMRT plans. Statistically significant differences between IMRT and RA data and between low and high energy groups have been observed and reported. Nevertheless, no constant trend was found to allow to rank one technique or one energy to be absolutely superior to all others for all parameters analyzed. The second objective was to assess the potential logistic benefit from the application of RapidArc with respect to IMRT. From both treatment time and number of fields involved, RapidArc proved a clear superiority to IMRT. In fact, because of the size of the targets, most of the IMRT fields shall be split due to hardware limitations of the MLC, as also shown by Hong et al [8], prolonging delivery time. RA proved to be more efficient also than HT where delivery time resulted ~2-4 time faster depending on the study analysed (modulation factors and field sizes being the key factors for HT). From the clinical viewpoint, this reflects also into safer treatments for the patients with reduced risk of internal organs movement and also patients movement during prolonged time on the couch. Most of the studies on whole abdominal radiotherapy, are based on the need to use at least two isocentres to cover the cranial-caudal extension of the target volumes, normally exceeding 40 cm in length (HT mitigates this issue with the continuous movement of the couch during irradiation). From IMRT and RapidArc point of view, the usage of two isocentres is not detrimental provided that, as in the present study, the same x and y isocentres coordinates are kept and only the z (cranial-caudal displacement) is modified. An overlap region is generated to avoid any field matching. This allows also an easier quality assurance procedure with standard IGRT tools (and even 2D planar kV or MV orthogonal images) where eventual small errors in z are much easier to be detected. RapidArc plans were designed using three arcs distributed on two isocentres performing a simultaneous optimization of all three at the same time and introducing an overlap of about 5 cm between the superior (one arc) and inferior (two arcs) groups. The elimination of matching of junction lines between arcs (also because using different collimator angles the projected lines of the field edges of different fields describe different trajectories during the rotation) implies that PTV and OARs doses in the arc-overlapping region are accounted for by the optimization engine due to the simultaneous processing of all three arcs preventing undue under- or over-dosages.

The third objective was to assess the degree of agreement of delivered vs. computed dose distributions. RapidArc and IMRT proved a substantial equivalence in terms of GAI results confirming what already observed in previous investigations [26,27,33,34].

Some investigations have been performed in the past years to assess the role of IMRT, IMAT and HT for WAR. Additional file 1: Table S1 summarizes the main findings, compared to the present one, from the studies of Duthoy et al, Hong et al, Garsa et al, Rochet et al, Jamema et al [8-12,18]. Obviously, similar comparisons have to be looked with extreme caution since, different patient selection and more important differences in the outline of targets and organs at risk can induce major biases in the relative analysis. The most striking potential sources of bias are the huge difference in the extension of the target volumes and in the overlap between targets and OARs. As an example, in our investigation 1-1.5 cm of rim of kidneys and liver have been included in the target volume and therefore these volumes had to be covered by the dose prescribed to PTV. This, obviously have a detrimental impact on the dose reported for these organs which is not easy to account for data from independent studies where, possibly, different overlaps, if any, are considered. Another source of discrepancy between studies is the intrinsic definition of what an OAR is, e.g. the definition of the ''bones'' or ''bone marrow'' structures which directly affects the values reported (with huge differences appearing between the Jamema [18] and current study with respect to, e.g. Rochet or Hong [8,11,12] data.

With a relatively low prescription to PTV_WAR, the sparing of kidneys and liver, results from Additional file 1: table S1 comparing this study with the results of other investigations, suggest that RA enables, for this class of patients, better target coverage (in fact improved compared to Rochet et al [11,12]) than OAR sparing (particularly of Rochet et al.). A more neutral comparison can be performed between the present study and the one by Jamema et al [18] which can be better paired since based on the same volumes and dose prescriptions. From this comparison it results that target coverage is slightly superior for HT than for RA but likely not clinically significant (differently from what observed from the Rochet et al data where differences are much more favorable for RA); sparing of liver and bone marrow is slightly improved by RA while sparing of kidneys is equivalent between the two techniques.

A last remark is for respiratory induced motion management which is not accounted by RapidArc in its present form. In whole abdominal treatments, respiratory motion is strongly correlated with diaphragm motion which could be subject to tracking given its periodicity. Feasibility studies were performed and demonstrated the general potential of tracking for RapidArc [36] but clinical implementation is not yet available. This is different from what currently available for IMRT where respiratory gating is routinely used in several institutes, specially for breast treatments although abdominal motion might poorly correlate to chest displacement measured by Varian's respiratory gating tools. As a surrogate of respiratory gating, it was suggested [37] that treatment during mid-ventilation phase might be reasonable solution since this is the phase where targets can be "seen" by static beams for the longest time provided adequate margins are defined. The dosimetric uncertainties caused by diaphragm motion were therefore not accounted for in this study for neither IMRT nor RapidArc plans but were also not accounted for in most of the studies with IMRT or Helical Tomotherapy with the exception of Garsa et al [10].

## Conclusion

The study addressed a comparison of RapidArc and IMRT with fixed gantry for whole abdominal radiotherapy in patients affected by ovarian cancer. Aims of the study were met and in summary: i) RapidArc allows the generation of adequate dose distribution with high target homogeneity and sufficient sparing of organs at risk, compared to IMRT; ii) RapidArc offers clear logistic advantages over IMRT with potential significant clinical implications, on system throughput and on minimization of patient movements; iii) pre-treatment clinical dosimetry study confirmed high reliability of RapidArc delivery compared to IMRT and in line with other recent investigations; iv) treatments at lower energy, RapidArc of IMRT might be preferable given the similar dosimetric features of corresponding high energy approaches because of reduced risk of secondary cancer induction for long term survivors.

## Competing interests

Dr. L. Cozzi acts as Scientific Advisor to Varian Medical Systems and is Head of Research and Technological Development to Oncology Institute of Southern Switzerland, IOSI, Bellinzona.

## Authors' contributions

UM carried out the study conception and design and drafted the manuscript.

LC carried out the study conception and design and drafted the manuscript.

SJ performed data collection, RE performed data collection, DD performed data collection, RS performed data collection, AF performed data collection and analysis, GN performed data collection and analysis, AC performed data collection and analysis, EV performed data collection and analysis, SS performed data collection. All authors read and approved the final manuscript.

## Supplementary Material

Additional file 1 **Table S1**. Synopsis of some dosimetric findings from recent investigations on the potential role of IMRT or Tomotherapy or RapidArc on whole abdomen radiotherapy for ovarian cancer treatment. Click here for file
