# Supplementary material for: Whole abdomen radiation therapy in ovarian cancers: a comparison between fixed beam and volumetric arc based intensity modulation
Source: Radiat Oncol. 2010 Nov 15;5:106. doi: 10.1186/1748-717X-5-106 (PMC2994871; doi:10.1186/1748-717X-5-106)
Supplement: Additional file 1 — Table S1. Synopsis of some dosimetric findings from recent investigations on the potential role of IMRT or Tomotherapy or RapidArc on whole abdomen radiotherapy for ovarian cancer treatment. [file 1748-717X-5-106-S1.DOC]

Table S1. Synopsis of some dosimetric findings from recent investigations on the potential role of IMRT or Tomotherapy or RapidArc on whole abdomen radiotherapy for ovarian cancer treatment.

|  |  | This study | Jamema [18] | Duthoy et al [9] | Hong et al [8] | Garsa et al [10] | Rochet et al [11] | Rochet et al [12] |
| --- | --- | --- | --- | --- | --- | --- | --- | --- |
|  | Technique | RapidArc  (15MV) | HT | IMAT | IMRT  (15 MV) | IMRT with Resp Gating  (6 MV) | HT | IMRT step and shoot HT |
|  | Treatment time | 4.8±0.2 min | 16.2±1.3 min (2.5 cm field)  9.1±0.7 min (5.0 cm field) | 13.8 min | - | 40-45 min | 18.1 min | IMRT: 37-38 min  HT: 19. min |
|  | Arcs/fields | 3 arcs | 1 helic, pitch 0.3 | 6-11 arcs | 5 fields | 5 fields | 1 helic, pitch 0.29 | IMRT: 9-12 fields  HT: 1 helic, pitch 0.29 |
|  | PTV volume | 6524±861 cm^3^ PTV_WAR  1211±158 cm^3^ PTV_Pelvis | 6270±470 cm^3^ PTV_WAR  1235±88 cm^3^ PTV_Pelvis | 8306 cm^3^ | 7935 cm^3^ | - | 7684 cm^3^ | 7684 cm^3^ |
|  | Dose Prescription | 25 Gy PTV_WAR  45 Gy PTV_Pelvis | 25 Gy PTV_WAR  45 Gy PTV_Pelvis | 33 Gy | 30 Gy | 30 PTV_WAR  44.4 PTV_Pelvis | 30 Gy | 30 Gy |
| PTV | V_90%_ [%] | 98.6±0.9 PTV_WAR  99.5±0.3 PTV_Pelvis | - | 89.9±5.7 | - | 100 PTV_WAR | 93.1 | 90.2±9.6 |
|  | V_95%_ [%] | 92.7±2.4 PTV_WAR  94.0±2.1 PTV_Pelvis | 95.6±2.7 PTV_WAR  95.7±2.4 PTV_Pelvis | 82.2±6.5 | 83.5±3.9 | - | 86.9 | 80.8±10.3 |
| Kidney | D_mean_ [Gy] | 16.0±1.8 Both  10.3±0.9 Both–PTV | 10.8±0.8 Both–PTV | 13.6±3.9 Right  16.1±3.6 Left | 16.1±0.4 | 16.1 | 9.8 Right  9.1 Left | 10.4±1.7 Right  9.4±1.5 Left |
| Liver | D_mean_ [Gy] | 21.8±0.9 all  14.8±0.5 normal | 25.2±0.8 all  18.3±1.4 normal | 24.4±6.3 | 28.5±0.3 | 30.1 | 21.6 Gy | 22.9±2.2 Gy |
| Bone Marrow | D_mean_ [Gy] | 22.6±1.5 | 24.1±1.6 | - | 18.5±1.0 | 25.0 | 10.6 | 12.1±1.7 |
